# Autoimmune activation as a determinant of atrial fibrillation among Turks: A prospective evaluation

**DOI:** 10.1097/MD.0000000000011779

**Published:** 2018-08-03

**Authors:** Barış Şimşek, Servet Altay, Nazmiye Özbilgin, Altan Onat

**Affiliations:** a Section of Cardiology, Siyami Ersek Center for Cardiovascular Surgery, Istanbul; b Department of Cardiology, Faculty of Medicine, Trakya University, Edirne; c Department of Cardiology, Cerrahpaşa Medical Faculty, Istanbul University, Istanbul, Turkey.

**Keywords:** apolipoprotein B, atrial fibrillation, autoimmune activation, low-density lipoprotein-cholesterol, sex hormone binding globulin

## Abstract

Although low-grade inflammation has been linked to the prediction of atrial fibrillation (AF), evidence from some reports suggest that autoimmune activation might potentially be a relevant mechanism. We assessed the predictive value of inflammation and other markers for the risk of incident AF.

A score of age-controlled anthropometric, lipid, and nonlipid variables was compared in participants with recorded nonvalvular persistent/permanent AF (n = 110) to those of a nested cohort sample (n = 1126) of the Turkish Adult Risk Factor study. Available values preceding by 2 (±1) years the development of AF were used regarding incident AF (n = 87) in multivariable regression.

Comparing age-controlled inflammation and other markers across the 2 groups, low apolipoprotein (apo) B and total cholesterol levels differed highly significantly in each sex. Moreover, low-density lipoprotein (LDL)-cholesterol and fasting insulin concentrations were significantly lower, sex hormone binding globulin (SHBG), glucose and systolic blood pressure higher in women alone, while C-reactive protein levels were similar. A model of multivariable logistic regression analyses for overall AF and 2 models for incident AF demonstrated a consistent inverse predictive value for apoB in each gender [relative risk (RR) 0.44 (95% confidence interval (CI), 95% CI 0.30–0.66], along with age, as main determinants. SHBG in females and waist circumference in males were further significantly associated with initial AF. Never smoking (compared with ever smoking) tended to predict AF.

These findings, collectively, are highly consistent with an autoimmune process in which damaged epitope of apoB due to proinflammatory state emerge as a basic mechanism in the development of AF. ApoB level is likely only apparently reduced due to partial escape from assay.

## 1 Introduction

Diverse cardiac or metabolic conditions and lifestyle changes beyond age are associated with atrial fibrillation (AF).^[1–3]^ Disturbances that promote ectopic firing and reentrant mechanisms with or without dilatation and fibrosis of the atrial myocardium are considered the basic underlying pathological anatomic substrate. This is rendered primarily by conditions inducing myocardial hypertrophy and heart failure. Obesity is a risk factor such that the corrected risk of incident AF increased by 28% corresponding to each 5 units of body mass index (BMI).^[4]^ Increase in systemic low-grade inflammation has been also linked to the prediction of AF, but C-reactive protein (CRP) and fibrinogen did not improve predictive ability independent of established clinical risk factors.^[5,6]^ The role of atherogenic dyslipidemia for the risk of AF has been uncertain, though in the combination of 2 cohorts, high triglycerides and low high-density lipoprotein (HDL) cholesterol were found to be independently predictive of AF.^[7]^ These constituents of atherogenic index of plasma attest to the operation of enhanced low-grade inflammation.^[8,9]^ To the surprise of some researchers, high levels of low-density lipoprotein (LDL) and total cholesterol were associated with a lower risk of AF in community-based studies.^[9–13]^ However, evidence exists that this constellation may represent autoimmune activation, a potential relevant mechanism (as explained below).

Despite a paradoxical appearance of LDL and total cholesterol results, their low levels have been known to precede the development of rheumatoid arthritis,^[14]^ as have low lipoprotein[Lp](a) concentrations preceded incident type-2 diabetes.^[15,16]^ Susceptibility to inverse associations of Lp(a) with the development of cardiac, metabolic, or rheumatic diseases involves population segments prone to impaired glucose tolerance. South Asian and Middle East populations, including Turkish adults, as well as Hispanic American people are typical examples.

We aimed to study epidemiologically the basic mechanism underlying the substrate changes for AF in a nested case–cohort design, specifically whether pro-inflammatory state and autoimmune activation, possible underlying chronic disease such as metabolic syndrome, type 2 diabetes, coronary heart disease, sleep disorders, and attention deficit in Turkish adults,^[17–19]^ contributes importantly to the development of AF. Several biomarkers little studied thus far in the prediction of AF were available in the Turkish Adult Risk Factor (TARF) study participants, and we examined the role of biomarkers that preceded incident AF by a limited period.

## 2 Methods

### 2.1 Study participants

Enrolled in the study were all cases of persistent AF recorded in the TARF surveys since the initial survey in 1990 (cases: 110 participants). A control cohort at an approximately 1:10 proportion in the same periods with similar sex distribution and relatively close age, consisting of 1126 individuals (568 men and 558 women), was recruited for comparison (just under one-half of the available participants). The baseline in the cohort was formed in 62.3% by the survey 1997–1998, while 75% participated in 2000 and latest in 2008. Participants younger than 35 years (n = 290 of 2810) were excluded to reduce the age gap, and those having available nearly all traditional cardiovascular risk markers were included. In subjects with AF, the initial year in which this arrhythmia was registered was taken as reference. This design allowed the prospective assessment of diverse variables predicting incident AF. Participants and methods on the population-based TARF study and details of the new cohort had previously been reported.^[20]^ The study was approved by the Ethics Committee of the Istanbul University Medical Faculty. Written informed consent was obtained from all participants.

### 2.2 Measurements of risk variables

Blood pressure (BP) was measured in the seated position on the right arm using an aneroid sphygmomanometer (Erka, Bad Tölz, Germany), after 5 minutes of rest, and the mean of 2 recordings was computed. Waist circumference was measured with the subject standing at the end of gentle expiration at the level midway between the lower rib margin and the iliac crest. Status of cigarette smoking at baseline was categorized into current smokers, former smokers, and never smokers. Physical activity was graded by the participant himself into 4 categories of increasing order (grades 1 to 4) with the aid of a scheme.^[21]^

Fasting was defined when blood drawing in individuals was 11 hours or more after the last meal. Serum concentrations of total cholesterol, fasting triglycerides, glucose, and HDL-cholesterol (directly without precipitation) were determined using enzymatic kits from Roche Diagnostics (Mannheim, Germany) on Roche-Hitachi Modular P800 autoanalyzer. Concentrations of fasting insulin, sex hormone binding globulin (SHBG), and total testosterone were determined by the electrochemiluminescent immunoassay method using Roche kits and Cobas e411 analyzer (Roche Diagnostics, Mannheim, Germany). Concentrations of serum Lp(a), apoA-I apoB, and CRP were measured by kits and nephelometry of Siemens Healthcare Diagnostic Products (Marburg, Germany). Plasma fibrinogen was assayed by the modified Clauss method using Fibrintimer II coagulometer and Multifibren U kit (Siemens Healthcare Diagnostics, Germany). Homeostatic model assessment (HOMA) was calculated with the following formula: insulin (mIU/L)∗ glucose (in mmol/L)/22.5.

### 2.3 Definitions

Hypertension denoted a systolic BP ≥140 mm Hg and/or diastolic BP ≥90 mm Hg, or use of antihypertensive medication. Individuals with metabolic syndrome were identified when 3 of the 5 criteria of the joint conference^[22]^ were met, modified for male abdominal obesity using as cutpoint ≥95 cm, as assessed in the TARF study.^[23]^ Individuals with diabetes were diagnosed with criteria of the American Diabetes Association,^[24]^ namely when plasma fasting glucose was ≥7 mmol/L (or 2-hour postprandial glucose >11.1 mmol/L) and/or the current use of diabetes medication.

AF was defined by absence of P-wave, presence of f-waves irregular in shape, size, and interval and at a frequency of 350 to 600/min (lead V1 was especially searched for fine fibrillation), and absolute irregularity of the QRS complexes.^[1]^ Electrocardiographies (ECGs) were recorded in each biennial survey. Persistent AF denoted existence of AF either in 2 consecutive survey recordings or, less frequently, when participant declared knowledge of its presence in the preceding 6 months or more, added to 1 recording.

### 2.4 Variables examined for and the baseline values preceding the development of AF

Age, sex, BP, waist circumference, smoking status, fasting glucose, lipids, lipoproteins and apolipoproteins, CRP, and sex hormones were investigated in relation to the development of AF. Baseline examination for anthropometric and biochemical values were considered those that preceded incident AF by a median 2 years [interquartile range (IQR) 1–4 years], with the purpose of obtaining information on low-grade inflammation and autoimmune processes potentially related to the AF incidence. For prevalent AF, values of the survey in which AF was first recorded constituted the baseline. Estimated marginal mean values were used to control for differences from the control cohort in age and sex.

### 2.5 Data analysis

Descriptive parameters were shown as means [± standard deviation (SD)]. Due to skewed distribution, geometric means were used uniformly for triglycerides, CRP, insulin, SHBG, testosterone, and Lp(a) values. In biennial surveys, baseline in the cohort and in participants with AF was formed as described in the Results. Two-sided *t* tests and Pearson Chi-square tests were used to analyze the differences between means and proportions of groups. Estimated marginal mean values were tested with paired method and adjustment for Bonferroni comparisons. A *P* value *<*.05 was considered as significant.

### 2.6 Inclusion criteria of model

We considered it appropriate to add variables that are significant in univariate analysis and associated with AF in the literature. Multivariable adjusted associations were assessed in logistic regression analyses for predicting AF whereby likelihood estimates [odds ratio (OR)] and 95% confidence intervals (95% CIs) were obtained. A value of *P* < .05 on the 2-tail test was considered statistically significant. Statistical analyses were performed using SPSS 10 (IBM) for Windows.

## 3 Results

Since the beginning of the TARF study in 1990, recorded nonvalvular persistent AF cases numbered 110 of which 87 met the criteria of incident AF. Mean age was 67.6 ± 12.3 years (Table 1). Incident AF was registered initially between 1993 and 2014 years, median in May 2006 (IQR 2000–2014). Although outcome of AF was beyond the scope of this study, a follow-up ranging from 1 to 16 years, median 24 months (IQR 20–72 months), was available in 57 of these individuals, during which AF persisted.

**Table 1:**
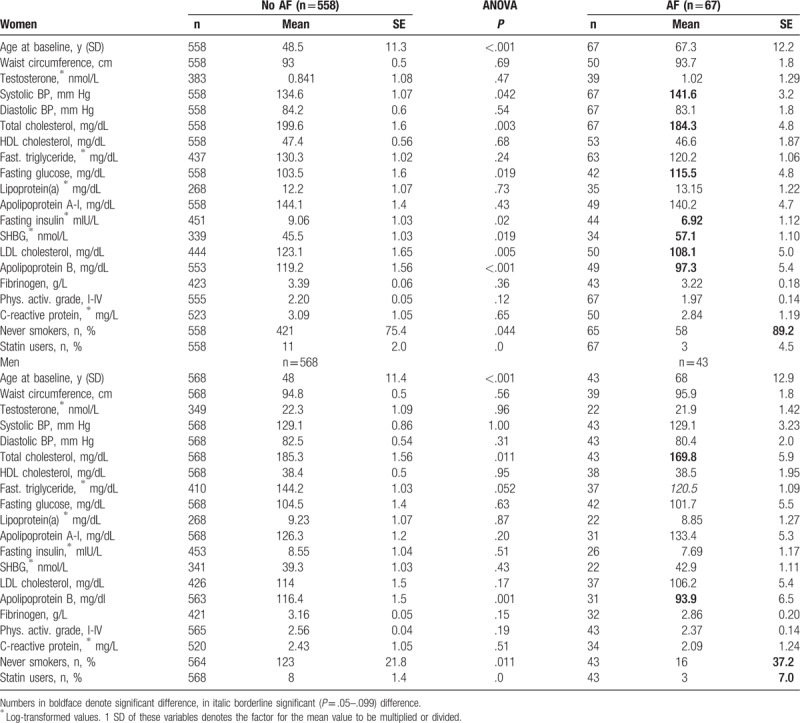
Estimated marginal means (SE) of variables at baseline of participants without atrial fibrillation (AF) and those subsequently developing AF.

The cohort to be compared consisted of 1126 participants at a mean 48 (± 11.3) years of age (Table 1). Their entry to the study averaged at mid-year 2000. They presented a follow-up of 0 to 16 years; mean 9.0 ± 5.25 years, median 9 years (IQR 4–14 years), making up a total follow-up of 10,130 person-years.

Estimated marginal means (SE) of 18 variables at baseline of participants without AF and those subsequently developing AF are summarized in Table 1. In cases of incident AF values preceding by 2 (±1) years, the development of AF was used. Both in the male and female patients with AF, apoB (by 19%) and total cholesterol (by 8%) were significantly lower than in the control sample. Women with AF additionally had significantly low LDL-cholesterol and fasting insulin (by 24%) levels, and significantly high systolic BP and SHBG levels, the latter amounting to 25% and 9% in women and men, respectively. Although fasting triglycerides were low in each gender, this reached borderline significance (*P* = .052) in men alone (by one-sixth). Of interest was the similarity in each group of waist circumferences, HDL-cholesterol (and in men fasting insulin and glucose) as metabolic syndrome (MetS) components, as well as of apoA-I, Lp(a), CRP, fibrinogen, and total testosterone levels. Never smokers were substantially higher in males and females with AF.

These findings were used in 3 models of multiple logistic regression analyses with the aim of investigating independent predictors. In the first model, 7 variables including age were examined (Table 2). Apo B decrements and aging displayed ORs at similar magnitudes of significant independent associations with the likelihood of AF in each sex; male participants using statins did so as well.

**Table 2:**
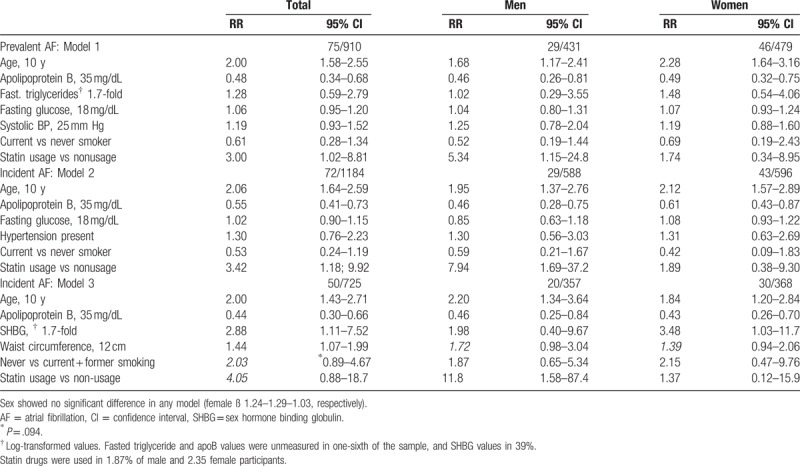
Multivariable logistic regression analyses of baseline variables in predicting prevalent or incident atrial fibrillation, by gender.

Two models were applied for incident AF. Model 2 comprised the nonsignificant hypertension [RR 1.30 (95% GA 0.76; 2.23)] replacing systolic BP and triglycerides. Significance of the 3 parameters persisted in similar magnitudes in RR. In model 3, beyond apoB and age, high SHBG in women, and wide waist circumference in combined sexes were significant independent predictors of incident AF. Current (compared with never) smoking, alike in other models, tended to protect (by one-half) against the development of AF albeit not attaining significance.

## 4 Discussion

We performed a nested case–control study from the cohort of the longitudinal TARF study for records of persistent/permanent AF. Incident AF cases were primarily investigated. Various anthropometric, lipid, and nonlipid parameters forming baseline values of participants with incident AF preceding AF by 2 (±1) years were compared sex-specifically with baseline values of the cohort. In logistic regression models, factors were investigated predicting AF significantly and independently. We obtained novel information. The 2 major independent predictors of AF, age and low serum apoB, were valid in each sex. In addition, elevated SHBG in women and use of statin drugs in men were significant predictors and never smoking was also at borderline significance in combined gender. These findings suggest that the basic mechanism underlying AF may be an autoimmune process encompassing dysfunction of protective proteins together with damaged apoB on Lp(a). Sex-specific result observations for statin use may be due to higher rates of statin use in men in our country, as well as ethnicity-specific sex-dependent autoimmune activation.

### 4.1 Reports on low cholesterol levels heralding the development of AF

It is an accepted fact in the literature that high LDL and total cholesterol are associated with an increased risk of atherosclerotic cardiovascular disease.^[25,26]^ But there are also supporting data for our study in the literature about AF and LDL-cholesterol inverse association. Two Japanese cohorts exhibited lower AF risk associated with high LDL- and total cholesterol.^[9,10]^ Lower AF risk for high serum total cholesterol was confirmed in the Cardiovascular Health Study.^[11]^ In participants from the Women's Health Study,^[12]^ 795 events of incident AF were remarkably predicted inversely by LDL-cholesterol (HR 0.72), driven by the number of small LDL and small very LDL particles. This suggests that low LDL-cholesterol may herald the development of AF.

### 4.2 Comments on current main findings

The most striking finding in each sex was reduced values (by about 19%) of serum apoB preceding the development of AF by a median 2 years, and this determinant predicted the development of AF independent of age, systolic BP, triglycerides, glucose, and smoking status. We shall expand our comments to this as well as to other findings further below. Multivariable adjusted apoB levels were evaluated to our knowledge only in the Women's Health Study^[12]^: a nonsignificant inverse association (HR 0.85) with AF was observed, more prominent in the upper 2 apoB quintiles.

We found lower age-adjusted fasting insulin levels in participants who were to develop AF than the control cohort, at a significant level in women alone. Although this may seem surprising, a similar inverse association has recently been reported in men (contrasted to women) from the Malmö Preventive Project with a long follow-up.^[27]^ Of 983 cases of incident AF, this inverse relationship was strong in normoglycemic men, weaker in subjects with impaired glucose tolerance, and positive in subjects with impaired fasting glucose. The authors considered that the relation between insulin and incident AF risk may be dependent on glucose regulation. Our findings are consistent with circulating Lp(a) being here the major confounder, in view of the inverse relationship between serum Lp(a) and fasting insulin^[16,28]^ and because impaired fasting glucose (IFG) status in nondiabetic people without MetS is associated with a less adverse cardiovascular risk profile and reduced future coronary heart disease (CHD) risk.^[28,29]^ Thus, low insulinemia may be a mediator of higher Lp(a) concentrations, or vice versa.

There is evidence of randomized trials to support a statin-associated reduction in CHD risk.^[25,30]^ Also, there are publications in the current literature that statin therapy has a general protective effect against AF.^[31]^ A seemingly paradoxal finding was the prediction of incident AF in males using statin drugs over prolonged periods. However, the TARF study also documented that appropriately instituted statin therapy in population subsets prone to MetS or enhanced low-grade inflammation may increase CHD risk in a primary prevention setting.^[32]^ Excess risk imparting may be attributed to a modifying effect of statins on Lp(a). In analogy to the current finding, antihypertensive drug use was reported in the Dutch PREVEND study as a highly significant determining variable of incident AF, using age- and sex-adjusted Cox regression models.^[33]^ Population-attributable risk estimates of reversible factors were stated to be led by antihypertensive drug use, greater than the aggregate of the next 3 factors (previous myocardial infarction or stroke and obesity).

Smoking is an established cause of a plethora of diseases and is responsible for 50% of all avoidable deaths in smokers, half of these due to CVD. The 10-year fatal CVD risk is approximately doubled in smokers.^[34]^ Also, there are studies that smoking increases the frequency of AF.^[35]^ A nearly 2-fold, albeit nonsignificant, future AF risk tended to be associated in never compared with ever smokers regardless of sex. This paradoxical finding is consistent with various reports of the TARF as well as with some findings reported elsewhere. The risk of development of diabetes was observed to be nearly halved among smoking compared with never smoking Turkish adults.^[36]^ This finding was subsequently confirmed among Japanese men^[37]^ and the HUNT study on Norwegian men and women.^[38]^ Moreover, in a large cross-sectional study on MetS in Polish women, MetS was detected more frequently in women having discontinued smoking.^[39]^ We concur in the TARF study^[17,40]^ with the opinion expressed in the HUNT study that active smoking inhibits the autoimmune process inducing type-2 diabetes in susceptible subjects.

### 4.3 Findings represent underlying autoimmune activation

Current main findings of “paradoxical” lower total and LDL-cholesterol levels in sex- and age-adjusted patients with AF, low serum apoB and high SHBG as main determinants of incident AF –beyond age – strongly support the hypothesis that autoimmune activation is the mechanism underlying the development of persistent AF. Lp(a) protein and its component apoB are presumably oxidatively damaged and aggregated to SHBG to form a complex. Circulating oxLDL/beta2-glycoprotein I complexes have been detected and assessed in autoimmune-mediated atherosclerosis.^[41]^ Such complexes comprising Lp(a) have been reported in association with premature atherosclerosis in patients with rheumatoid arthritis^[42]^ and coronary artery disease.^[43]^

Worth emphasizing is that (by about 10%) lower serum levels of total and LDL-cholesterol and fasting triglycerides were each significant covariates of age- and sex-adjusted prevalent and incident AF. Together with low apoB and other findings, the mentioned lipid findings suggest the association with autoimmune processes. The AF findings in the above-stated Women's Health Study,^[12]^ in whom diabetes was predicted as well by low Lp(a),^[15]^ support this.

An intriguing finding is the SHBG elevation in cases of AF in women, reaching significance in women. Low SHBG levels have been recognized to generally raise cardiometabolic risk in the TARF^[44]^ as well as in other studies.^[45]^ Although we have been unable to find a report on elevated SHBG as a risk factor, in an as TARF study SHBG, protecting against “hypertriglyceridemic waist” phenotype in women, elevated SHBG paradoxically conferred marked CHD risk.^[46]^ This may be attributed to proinflammatory conversion of the SHBG when aggregated to serum apoB, an oxidatively damaged protein.

In a previous TARF study,^[47]^ serum CRP in males with AF was significantly lower than both CRP levels in men without AF and in females with AF. Age-adjusted CRP levels in the current work in each sex were, likewise, lower (by about one-tenth) in AF patients without reaching significance.

### 4.4 Can the proposed mechanism lead to AF?

How can the mechanism of autoimmune activation in a milieu of chronic enhanced inflammation induce an electrophysiologic substrate for AF, which represents a final common pathway for multiple predisposing conditions?^[3]^ Following considerations appear plausible. The requirement of substantial fibrosis of the right atrium may well be a consequence of autoimmune activation, analogous to the gradual development of fibrosis in chronic steatohepatitis. Prolonged BP elevation mediated by the autoimmune process^[48]^ may also contribute to myocardial hypertrophy and to atrial fibrosis leading to incident AF. In the future, 3-dimensional speckle tracking echocardiography may be helpful in detecting atrial fibrosis as well as ventricular remodeling.^[49]^ And new technologies can be used to follow atrial arrhythmia.^[50,51]^ Endothelial dysfunction, a common occurrence in autoimmune activation and proinflammatory state, may facilitate the appearance of re-entries secondary to fibrosis-based conduction disturbance of regular sinus impulses.^[52]^

### 4.5 Strength and limitations

Measuring parameters usually not investigated thus far in the prediction of AF forms a major strength. Using as baseline values preceding incident AF by only a few years is a further advantage. The longitudinal nested case–cohort design allowed the emergence of significant novel findings, despite the relatively limited number of subjects with AF. The compared cohort was substantially younger than subjects developing AF, a limitation that relatively persists despite being reduced by age-adjustment, but inverse association of AF with apoB-containing lipoproteins, one of the major findings, had been reported previously by other workers. Recording biennial ECG is a further limitation rendering lack of capture of some of the studied persistent arrhythmia. The absence of all cardiovascular drug information is another limitation. Validity or applicability of findings may be limited for populations or population segments not prone to enhanced low-grade inflammation, glucose intolerance, or MetS.

## 5 Conclusion

In prospective nested case–control analysis with middle-aged and elderly participants drawn from the TARF study, we confirmed previous reports of “paradoxical” relatively low total and LDL-cholesterol levels in sex- and age-adjusted patients with AF. Low serum apoB was a main determinant of incident AF in each sex, along with higher SHBG, age, and wider waist girth, as did never smokers tend so. Presence of hypertension and increase in triglycerides failed to reach significance. Elicited findings support the notion of enhanced proinflammatory state coupled with an autoimmune process in which apoB, presumably aggregated to SHBG, emerge as a basic mechanism in the development of AF in the elderly; this is alike the dynamics for type-2 diabetes and inflammatory rheumatic disease.

## Author contributions

**Conceptualization:** Servet Altay, Nazmiye Özbilgin, Altan Onat.

**Data curation:** Barış Şimşek.

**Formal analysis:** Barış Şimşek, Servet Altay, Altan Onat.

**Funding acquisition:** Barış Şimşek.

**Investigation:** Barış Şimşek, Servet Altay.

**Methodology:** Barış Şimşek, Servet Altay, Nazmiye Özbilgin.

**Project administration:** Altan Onat.

**Resources:** Barış Şimşek, Nazmiye Özbilgin.

**Supervision:** Altan Onat.

**Validation:** Nazmiye Özbilgin.

**Visualization:** Altan Onat.

**Writing – original draft:** Servet Altay, Altan Onat.

Author name: orcid number

## References

[1] Fuster V Rydén LE Cannom DS European Heart Rhythm Association; Heart Rhythm Society. ACC/AHA/ESC 2006 Guidelines for the management of patients with atrial fibrillation: a report of the American College of Cardiology/American Heart Association Task Force on Practice Guidelines and the European Society of Cardiology Committee for Practice Guidelines (Writing Committee to Revise the 2001 Guidelines for the Management of Patients With Atrial Fibrillation) European Heart Rhythm Association and the Heart Rhythm Society. J Am Coll Cardiol 2006;48:854–906. 16904574 10.1016/j.jacc.2006.07.009

[2] Chung MK Martin DO Sprecher D C-reactive protein elevation in patients with atrial arrhythmias: inflammatory mechanisms and persistence of atrial fibrillation. Circulation 2001;104:2886–91. 11739301 10.1161/hc4901.101760

[3] Andrade J Khairy P Dobrev D The clinical profile and pathophysiology of atrial fibrillation: relationships among clinical features, epidemiology and mechanisms. Circ Res 2014;114:1453–68. 24763464 10.1161/CIRCRESAHA.114.303211

[4] Dublin S French B Glazer NL Risk of new-onset atrial fibrillation in relation to body mass index. Arch Intern Med 2006;166:2322–8. 17130384 10.1001/archinte.166.21.2322

[5] Altay S Çakmak HA Karadeniz FO Atrial Fibrilasyon Hastalarında Yüksek Plazma Pentraxin 3 Düzeyi: İnflamatuar Sürecin ve Rekürrensin Olası Göstergesi. MN Kardiyoloji 2016;23:1–6.

[6] Schnabel RB Larson MG Yamamoto JF Relations of biomarkers of distinct pathophysiological pathways and atrial fibrillation incidence in the community. Circulation 2010;121:200–7. 20048208 10.1161/CIRCULATIONAHA.109.882241 PMC3224826

[7] Alonso A Yin X Roetker NS Blood lipids and the incidence of atrial fibrillation: the Multi-ethnic Study of Atherosclerosis and the Framingham Heart Study. J Am Heart Assoc 2014;3:e001211. 25292185 10.1161/JAHA.114.001211 PMC4323837

[8] Onat A Can G Kaya H Atherogenic index of plasma” (log10 triglyceride/high-density lipoprotein-cholesterol) predicts high blood pressure, diabetes, and vascular events. J Clin Lipidol 2010;4:89–98. 21122635 10.1016/j.jacl.2010.02.005

[9] Iguchi Y Kimura K Shibazaki K Annual incidence of atrial fibrillation and related factors in adults. Am J Cardiol 2010;106:1129–33. 20920652 10.1016/j.amjcard.2010.06.030

[10] Watanabe H Tanabe N Yagihara N Association between lipid profile and risk of atrial fibrillation. Circ J 2011;75:2767–74. 21914959 10.1253/circj.cj-11-0780

[11] Lopez FL Agarwal SK Maclehose RF Blood lipid levels, lipids lowering medications, and the incidence of atrial fibrillation: the Atherosclerosis Risk in Communities Study. Circ Arrhythm Electrophysiol 2012;5:155–62. 22227953 10.1161/CIRCEP.111.966804 PMC3290134

[12] Mora S Akinkuolie AO Sandhu RK Paradoxical association of lipoprotein measures with incident atrial fibrillation. Circ Arrhythm Electrophysiol 2014;7:612–9. 24860180 10.1161/CIRCEP.113.001378 PMC4591535

[13] Kara K Geisel MH Möhlenkamp S B-type natriuretic peptide for incident atrial fibrillation: the Heinz Nixdorf Recall study. J Cardiol 2015;65:453–8. 25241013 10.1016/j.jjcc.2014.08.003

[14] Myasoedowa E Crowson CS Kremers HM Total cholesterol and LDL levels decrease before rheumatoid arthritis. Ann Rheum Dis 2010;69:1310–4. 19854708 10.1136/ard.2009.122374 PMC2890039

[15] Mora S Kamstrup PR Rifai N Lipoprotein(a) and risk of type-2 diabetes. Clin Chem 2010;56:1252–60. 20511445 10.1373/clinchem.2010.146779 PMC2912456

[16] Onat A Çoban N Can G Low “quotient” Lp(a) concentration mediating autoimmune activation predicts cardiometabolic risk. Exp Clin Endocr Diabetes 2015;123:11–8. 10.1055/s-0034-1385922 25314652

[17] Onat A Can G Enhanced proinflammatory state and autoimmune activation: a breakthrough to understanding chronic diseases. Curr Pharm Design 2014;20:575–84. 10.2174/138161282004140213145551 23565630

[18] Araz Altay M Sleep disorders and attention deficit: a consequence of proi̇nflammatory state? J Clin Sleep Med 2018;14:1081. 29852912 10.5664/jcsm.7190 PMC5991949

[19] Altay MA Does attention deficit hyperactivity disorder have arrhythmia potential? Anadolu Psikiyatri Derg 2018;May 266. Doi: 10.5455/apd.294213.

[20] Onat A Risk factors and cardiovascular disease in Turkey. Atherosclerosis 2001;156:1–0. 11368991 10.1016/s0021-9150(01)00500-7

[21] Onat A Şenocak M Örnek E Plasma lipids and their interrelation in Turkish adults. J Epidem Commun Health 1992;46:470–6. 10.1136/jech.46.5.470 PMC1059634 1479313

[22] Expert Panel on Detection, Evaluation, and Treatment of High Blood Cholesterol in Adults. Executive Summary of the Third Report of the National Cholesterol Education Program (NCEP) Expert Panel on Detection, Evaluation, and Treatment of High Blood Cholesterol in Adults (Adult Treatment Panel III). JAMA 2001;285:2486–97. 11368702 10.1001/jama.285.19.2486

[23] Onat A Uyarel H Hergenç G Determinants and definition of abdominal obesity as related to risk of diabetes, metabolic syndrome and coronary disease in Turkish men: a prospective cohort study. Atherosclerosis 2007;191:182–90. 16678831 10.1016/j.atherosclerosis.2006.03.012

[24] Genuth S Alberti KG Bennett P Expert Committee on the Diagnosis and Classification of Diabetes Mellitus. Follow-up report on the diagnosis of diabetes mellitus. Expert Committee on the Diagnosis and Classification of Diabetes Mellitus. Diabetes Care 2003;26:3160–7. 14578255 10.2337/diacare.26.11.3160

[25] Stone NJ Robinson JG Lichtenstein AH 2013 ACC/AHA guideline on the treatment of blood cholesterol to reduce atherosclerotic cardiovascular risk in adults: a report of the American College of Cardiology/American Heart Association Task Force on Practice Guidelines. Circulation 2014; 129:1–45.

[26] Nordestgaard B Chapman M Ray K Lipoprotein(a) as a cardiovascular risk factor: current status. Eur Heart J 2010;31:2844–53. 20965889 10.1093/eurheartj/ehq386 PMC3295201

[27] Johnson LSB Juhlin T Engström G Low fasting plasma insulin is associated with atrial fibrillation in men from a cohort study – the Malmö Preventive Project. BMC Cardiovasc Disord 2014;14:107. 25150967 10.1186/1471-2261-14-107 PMC4236524

[28] Onat A Aydın M Can G Impaired fasting glucose: pro-diabetic, “atheroprotective” and modified by metabolic syndrome. World J Diabetes 2013;4:210–8. 24147205 10.4239/wjd.v4.i5.210 PMC3797886

[29] Altay S Onat A Karadeniz Y Prediction by low plasma HbA1c of mortality, cardiac and noncardiac disease risk: modulation by diabetic status and sex. J Investig Med 2015;63:821–7. 10.1097/JIM.0000000000000216 26107424

[30] Catapano AL Graham I De Backer G 2016 ESC/EAS Guidelines for the management of dyslipidaemias. Eur Heart J 2016;37:2999–3058. 27567407 10.1093/eurheartj/ehw272

[31] Sai C Li J Ruiyan M Atorvastatin prevents postoperative atrial fibrillation in patients undergoing cardiac surgery. Hellenic J Cardiol 2018;[Epub ahead of print]. 10.1016/j.hjc.2017.12.012 29307691

[32] Onat A Aydın M Köroğlu B Statin therapy and increased coronary heart disease risk in primary prevention of people with enhanced low-grade inflammation. Türk Klin J Cardiovasc Sci 2014;26:118–27.

[33] Vermond RA Geelhoed BH Verweij N Incidence of atrial fibrillation and relationship with cardiovascular events, heart failure, and mortality: a community-based study from the Netherlands. J Am Coll Cardiol 2015;66:1000–7. 26314526 10.1016/j.jacc.2015.06.1314

[34] Piepoli MF Hoes AW Agewall S 2016 European Guidelines on cardiovascular disease prevention in clinical practice: the Sixth Joint Task Force of the European Society of Cardiology and Other Societies on Cardiovascular Disease Prevention in Clinical Practice (constituted by representatives of 10 societies and by invited experts)Developed with the special contribution of the European Association for Cardiovascular Prevention & Rehabilitation (EACPR). European Heart Journal 2016;37:2315–81. 27222591 10.1093/eurheartj/ehw106 PMC4986030

[35] Chamberlain AM Agarwal SK Folsom AR Smoking and incidence of atrial fibrillation: results from the Atherosclerosis Risk in Communities (ARIC) study. Heart Rhythm 2011;8:1160–6. 21419237 10.1016/j.hrthm.2011.03.038 PMC3139831

[36] Onat A Özhan H Esen AM Prospective epidemiologic evidence of a “protective” effect of smoking on metabolic syndrome and diabetes among Turkish women – without associated overall health benefit. Atherosclerosis 2007;193:380–9. 16926017 10.1016/j.atherosclerosis.2006.07.002

[37] Nagaya T Yoshida H Takahashi H Heavy smoking raises risk for type 2 diabetes mellitus in obese men; but, light smoking reduces the risk in lean men: a follow-up study in Japan. Ann Epidemiol 2008;18:113–8. 18083537 10.1016/j.annepidem.2007.07.107

[38] Rasouli B Grill V Midthjell K Smoking is associated with reduced risk of autoimmune diabetes in adults contrasting with increased risk in overweight men with type 2 diabetes: a 22-year follow-up of the HUNT study. Diabetes Care 2013;36:604–10. 23172971 10.2337/dc12-0913 PMC3579345

[39] Kwasniewska M Pikala M Kaczmarczyck-Chalas K Smoking status, the menopausal transition, and metabolic syndrome in women. Menopause 2012;19:194–201. 22011755 10.1097/gme.0b013e3182273035

[40] Altay S Onat A Özpamuk-Karadeniz F Renal “hyperfiltrators” are at elevated risk of death and chronic diseases. BMC Nephrol 2014;15:160. 25278185 10.1186/1471-2369-15-160 PMC4190443

[41] Lopez LR Bruckner TR Hurley BL Determination of oxidized low-density lipoprotein (ox-LDL) versus oxLDL/beta2-GPI complexes for the assessment of autoimmune-mediated atherosclerosis. Ann N Y Acad Sci 2007;1109:303–10. 17785319 10.1196/annals.1398.036

[42] Zhang C Li X Niu D Increased serum levels of ß2-GPI-Lp(a) complexes and their association with premature atherosclerosis in patients with rheumatoid arthritis. Clin Chim Acta 2011;412:1332–6. 21473861 10.1016/j.cca.2011.03.029

[43] Wang J Gong J Li H Lipoprotein(a) complexes with beta2-glycoprotein I in patients with coronary artery disease. J Atheroscler Thromb 2012;19:81–9. 22056596 10.5551/jat.9340

[44] Onat A Hergenç G Karabulut A Serum sex hormone-binding globulin, a determinant of cardiometabolic disorders independent of abdominal obesity and insulin resistance in elderly men and women. Metabolism 2007;56:1356–62. 17884445 10.1016/j.metabol.2007.05.020

[45] Brand JS Rovers MM Yeap BB Testosterone, sex hormone-binding globulin and the metabolic syndrome in men: an individual participant data meta-analysis of observational studies. PLoS One 2014;9:e100409. 25019163 10.1371/journal.pone.0100409 PMC4096400

[46] Karagöz A Onat A Aydın M Distinction of hypertriglyceridemic waist phenotype from simple abdominal obesity: interaction with sex hormone-binding globulin levels to confer high coronary risk. Postgrad Med 2017;129:288–95. 27846754 10.1080/00325481.2017.1261608

[47] Uyarel H Onat A Yüksel H Incidence, prevalence, and mortality estimates for chronic atrial fibrillation in Turkish adults. Türk Kardiyol Dern Arş 2008;36:214–22. 18765964

[48] Onat A Can G Örnek E Increased apolipoprotein A-I levels mediate the development of prehypertension. Anadolu Kardiyol Derg 2013;13:306–14. 23591583 10.5152/akd.2013.106

[49] Xu L Huang X Ma J Value of three-dimensional strain parameters for predicting left ventricular remodeling after ST-elevation myocardial infarction. Int J Cardiovasc Imaging 2017;33:663–73. 28150084 10.1007/s10554-016-1053-3

[50] Zhang H Gao Z Xu L A meshfree representation for cardiac medical image computing. IEEE J Transl Eng Health Med 2018;6:1800212. 29531867 10.1109/JTEHM.2018.2795022 PMC5794334

[51] Xia Y Zhang H Xu L An automatic cardiac arrhythmia classification system with wearable electrocardiogram. IEEE Access 2018;6:16529–38.

[52] Castro-Faria-Neto HC Stafforini DM Prescott SM Regulating inflammation through the anti-inflammatory enzyme platelet-activating factor-acetylhydrolase. Mem Inst Oswaldo Cruz 2005;100 (suppl 1):83–91. 15962103 10.1590/s0074-02762005000900014

